# Lord Knutsford and Sir Victor Horsley

**Published:** 1914-06-27

**Authors:** 

**Affiliations:** Kneesworth Hall, Royston, Herts


### THE EDITOR'S LETTER-BOX.

### Lord Knutsford and Sir Victor Horsley.

*To the Editor of* THE HOSPITAL.

SIR,—I presume Sir Victor Horsley must have hoped that his after-dinner speech made at Birmingham after the recent "Stage Army" Conference would not be reported. But imagine the value to be attached to a man's statements who dares to say to his assembled admirers that "the opposition to Registration has dwindled down to the opposition of one man—Lord Knutsford!"

He knows quite well, because he has himself visited them, that the matrons of several of the largest hospitals in London are opposed—Brompton, King's, Middlesex, Royal National Orthopædic, St. Thomas's, St. George's, St. Mary's, the Metropolitan, and the Westminster. He knows that 224 matrons signed the protest against Registration when it was issued a few years ago. And he may be surprised to know, but at any rate I hope it will prevent his ever making such a silly statement again, that I have received protests against the present Bill signed by 340 matrons, 33 assistant matrons, and nearly 2,000 nurses—and this number will be increased.

It is true that I am perhaps the most active opponent who appears in public, just as he is the most active male advocate on the other side. Matrons are too busy, if they are doing their work, to spend their time in attending conferences and public meetings where they hear the same speakers over and over again.—Yours faithfully,

KNUTSFORD.

Kneesworth Hall, Royston, Herts,
June 22, 1914.

